# Retraction: A high-throughput metabolomics strategy for discovering the influence of differential metabolites and metabolic pathways of huaxian capsules on sepsis-associated Qi deficiency and blood stasis syndrome

**DOI:** 10.1039/d1ra90055b

**Published:** 2021-02-03

**Authors:** Laura Fisher

**Affiliations:** Royal Society of Chemistry Thomas Graham House, Science Park, Milton Road Cambridge CB4 0WF UK advances-rsc@rsc.org

## Abstract

Retraction of ‘A high-throughput metabolomics strategy for discovering the influence of differential metabolites and metabolic pathways of huaxian capsules on sepsis-associated Qi deficiency and blood stasis syndrome’ by Qun Liang *et al.*, *RSC Adv.*, 2019, **9**, 30868–30878, DOI: 10.1039/C9RA06679A.

The Royal Society of Chemistry hereby wholly retracts this *RSC Advances* article due to concerns with the reliability of the data. The images in the article were screened by an image integrity expert. Images presented in this article have been duplicated in another publication by the authors.^1^

Two of the kidney images (control group and model group) in Fig. 1 are identical to images presented in Fig. 2 of ref. 1, which represent different experiments.

Given the significance of the concerns about the validity of the data, the findings presented in this paper are not reliable.

Qun Liang does not agree with the retraction. The other authors were informed but have not responded to any correspondence regarding the retraction.

Signed: Laura Fisher, Executive Editor, *RSC Advances*

Date: 19^th^ January 2021

## Supplementary Material
